# Machine learning-based prediction model for chronic brucellosis: a multi-feature approach using clinical and laboratory data

**DOI:** 10.3389/fcimb.2025.1700233

**Published:** 2025-11-19

**Authors:** Rong Wang, Bin Niu, Chenming Zhang, Yinghan Wang, Xin Zhang, Haiyan Tian, Liaoyun Zhang

**Affiliations:** 1 Department of Infectious Diseases, The First Hospital of Shanxi Medical University, Taiyuan, China; 2 raduate School, Shanxi Medical University, Taiyuan, China; 3 Academy of Medical Sciences, Shanxi Medical University, Taiyuan, China

**Keywords:** brucellosis, chronic progression, machine learning, risk prediction, risk stratification

## Abstract

**Background:**

Chronic progression is a major clinical challenge in human brucellosis (HB), affecting nearly one-third of patients and leading to long-term disability. Reliable early prediction tools are lacking, hindering timely risk stratification and individualized management. This study aimed to develop and validate machine learning (ML) models to predict chronic progression using routinely available clinical and laboratory data.

**Methods:**

We retrospectively analyzed 555 patients with confirmed brucellosis admitted between 2019 and 2024. Clinical characteristics and laboratory indicators at admission were collected. Feature selection was performed using Boruta and recursive feature elimination. Six supervised ML models (random forest [RF], LightGBM, XGBoost, logistic regression [LR], multilayer perceptron [MLP], and support vector machine [SVM]) were constructed and evaluated by discrimination, calibration, clinical utility, and predictive metrics. Model interpretability was assessed using SHapley Additive exPlanations (SHAP), and a web-based prediction tool was developed.

**Results:**

Of 555 patients, 144 (25.9%) progressed to chronic brucellosis. Compared with the recovery group, chronic cases presented more frequently with arthralgia and arthritis and showed distinct biochemical profiles, including lower alanine aminotransferase (ALT), aspartate aminotransferase (AST), triglycerides (TG), and higher high-density lipoprotein cholesterol (HDL-C), albumin (ALB), blood urea nitrogen (BUN), and uric acid (UA). Among the six models, RF consistently demonstrated the most robust performance across metrics, achieving the highest AUC in the test set (0.782, 95% CI: 0.701 - 0.856), superior calibration (Emax = 0.155), and the greatest net clinical benefit in decision curve analysis. SHAP analysis identified TG, HDL-C, UA, eosinophil count, PA, ALT, BUN, and GLB as the most influential predictors, with biologically plausible associations.

**Conclusion:**

Using eight routinely available variables, the RF model demonstrated moderate discrimination with well-calibrated probability estimates but limited sensitivity. The tool may assist early risk stratification of chronic brucellosis when combined with clinical judgment; however, its predictive performance should be interpreted cautiously until validated in external, multicenter, and prospective studies.

## 1 Introduction

Brucellosis is one of the most prevalent zoonotic infections worldwide, caused by Brucella spp. and transmitted through direct contact with infected animals or the ingestion of unpasteurized animal products (Qureshi et al., 2023). Annually, an estimated 1.6 to 2.1 million new human brucellosis (HB) cases occur globally, although the true incidence is likely underestimated due to diagnostic delays and underreporting (Laine et al., 2023).

The disease remains endemic in regions such as the Middle East, Central Asia, South America, and China, where agricultural and pastoral practices facilitate ongoing transmission (Chen et al., 2023). In the Middle East and Central Asia, *B. melitensis* remains predominant, with recurrent outbreaks linked to pastoral exposure (Dean et al., 2012). In sub-Saharan Africa, incidence remains high—for instance, Kenya reports a national seroprevalence of 6.8% (95% CI: 6.2–7.4%) and community rates up to 84 per 100,000 person-years (Njeru et al., 2016). In South America, endemic transmission persists in Peru and Bolivia, mainly through occupational and foodborne exposure (Munyua et al., 2021). In China, surveillance shows a renewed rise, from 45,046 cases (3.25/100,000) in 2019 to 70,439 (4.99/100,000) in 2023, with Inner Mongolia exceeding 50 per 100,000 (Liu et al., 2025). Approximately 10–30% of patients progress to chronic or relapsing disease with musculoskeletal or neurologic involvement (Maduranga et al., 2024).

Clinically, brucellosis presents with diverse and nonspecific manifestations that vary between the acute and chronic phases. Acute brucellosis typically presents with fever, profuse sweating, hepatosplenomegaly, myalgia, and arthralgia, often mimicking other febrile or inflammatory illnesses (Liu et al., 2023). In contrast, chronic brucellosis is characterized by symptom persistence beyond six months, with predominant features including persistent fatigue, recurrent arthralgia, osteoarticular involvement (such as spondylitis and arthritis), and neuropsychiatric complications (Qureshi et al., 2023). These chronic manifestations can lead to long-term disability, markedly impairing quality of life and increasing healthcare burden.

Brucellosis can affect multiple organ systems, resulting in a wide spectrum of complications. Osteoarticular involvement is the most frequent, observed in up to 40–60% of cases, and includes spondylitis, arthritis, and sacroiliitis (Bosilkovski et al., 2004). Neurologic complications, collectively known as neurobrucellosis, include meningitis, encephalitis, brain abscess, and peripheral neuropathy, which may lead to lasting deficits (Gul et al., 2009). Cardiovascular involvement, particularly brucella endocarditis, is rare (<2% of cases) but accounts for the majority of brucellosis-related deaths (Vahabi et al., 2019). Hepatic, genitourinary, and cutaneous involvement have also been reported, further underscoring the systemic nature of this infection (Jin et al., 2023).

Despite growing recognition of disease chronicity, existing research has primarily focused on molecular distinctions between acute and chronic stages to improve diagnosis, with limited attention to prognostic modeling (Yang et al., 2023; Li et al., 2024). No validated clinical tools currently exist to predict the risk of chronic progression at the time of initial diagnosis, hindering early risk stratification and personalized intervention. While conventional diagnostic methods such as serology and culture remain essential for detection, they lack prognostic utility in forecasting chronic outcomes (Yagupsky et al., 2019).

In recent years, machine learning (ML) has emerged as a promising method for individualized disease risk prediction by leveraging high-dimensional clinical and laboratory data (Delpino et al., 2022). Several ML-based studies have demonstrated high diagnostic accuracy in identifying brucellosis cases at an early stage (Wang et al., 2023). However, these models have not addressed disease trajectory prediction, particularly the risk of chronic progression.

To fill this gap, the present study aimed to develop and validate an ML-based predictive model capable of identifying patients at risk of chronic brucellosis. We incorporated explainable artificial intelligence (AI) techniques to identify key features contributing to chronicity and compared the performance of multiple algorithms using comprehensive evaluation metrics, thereby establishing a clinically interpretable and robust predictive framework.

## 2 Materials and methods

### 2.1 Study population

This study enrolled 555 participants diagnosed with brucellosis at the First Hospital of Shanxi Medical University from May 2019 to December 2024. Baseline clinical and laboratory characteristics were collected for all participants.

The diagnosis followed the criteria of the national guideline “Diagnosis for Brucellosis (WS 269-2019)” issued by the National Health Commission in 2019 (National Health Commission of the People’s Republic of China, 2025):

1. Epidemiological exposure, such as close contact with livestock or animal products suspected of carrying Brucella, or ingestion of unpasteurized dairy or undercooked meat.
2. Clinical symptoms including prolonged fever (low- or high-grade), excessive sweating, fatigue, arthralgia, or myalgia, some patients had lymphadenopathy, hepatosplenomegaly, or testicular swelling, while a few exhibited various rashes or jaundice.
3. Laboratory findings, including positive results of the rose bengal plate agglutination test (RBT), colloidal gold immunochromatographic assay (GICA), and enzyme-linked immunosorbent assay (ELISA). In addition, Brucella organisms were observed by Gram staining of cultured isolates.

A clinical diagnosis required meeting criteria 1) and 2), together with any one of 3) simultaneously.

Patients were categorized into the recovery group or the chronic group according to whether clinical symptoms persisted after completing six months of standardized antimicrobial therapy. To reduce subjectivity, all outcome classifications were independently adjudicated by two experienced infectious disease physicians; any discrepancies were resolved through consensus with a third senior clinician.

The study protocol was approved by the Ethics Committee of the First Hospital of Shanxi Medical University (NO.KYYJ-2025-143). Follow-up information was obtained retrospectively through review of medical records and standardized telephone interviews. The requirement for consent for retrospective chart review was waived. This study adhered to the STROBE and TRIPOD reporting guidelines.

### 2.2 Candidate predictor variables

Clinical and demographic data, clinical characteristics, and laboratory variables at admission were retrospectively collected in this study, shown in **Table 1**.

**Table 1:** List of the features enrolled in the study.

Categories	Variables
Demographics	age, gender
Clinical Characteristics	
Symptoms	fever, myalgia, fatigue, anorexia, headache, arthralgia
Organ Involvement	hepatomegaly, splenomegaly, arthritis, neurobrucellosis, cardiac involvement in brucellosis, genitourinary involvement in brucellosis
Laboratory Variables	
Complete Blood Count	white blood cell count (WBC), red blood cell (RBC), hemoglobin concentration (HGB), platelet count (PLT), lymphocytes, neutrophils, monocytes, eosinophil, basophil, hematocrit (HCT), mean corpuscular volume (MCV), mean corpuscular hemoglobin (MCH), mean corpuscular hemoglobin concentration (MCHC), red cell distribution width-coefficient of variation (RDW-CV), red cell distribution width-standard deviation (RDW-SD), platelet distribution width (PDW), platelet-large cell ratio (P-LCR), plateletcrit (PCT-PLT), mean platelet volume (MPV)
Liver Function Tests	alanine aminotransferase (ALT), aspartate aminotransferase (AST), total protein (TP), albumin (ALB), globulin (GLB), prealbumin (PA), total bilirubin (TBil), direct bilirubin (DBIL), indirect bilirubin (IBIL), alkaline phosphatase (ALP), gamma-glutamyl transferase (GGT), total bile acid (TBA)
Renal Function Tests& Electrolytes	urea nitrogen (BUN), creatinine (CRE), uric acid (UA), potassium (K), sodium (Na), chloride (Cl)
Coagulation Function Tests	prothrombin activity (PT-S), prothrombin time (PT), international normalized ratio (INR), activated partial thromboplastin time (APTT), thrombin time (TT), d-dimer (D-D), fibrinogen concentration (FIB-C)
Blood Lipid Profile	total cholesterol (TC), triglycerides (TG), high-density lipoprotein cholesterol (HDL-C), low-density lipoprotein cholesterol (LDL-C)
Inflammatory Markers	erythrocyte sedimentation rate (ESR), procalcitonin (PCT), C-reactive protein (CRP), cyclic citrullinated peptide antibody (CCP)
Diagnostic Tests	blood culture, SAT

### 2.3 Treatment plan

All included patients were treated in strict accordance with the Diagnostic Criteria for Brucellosis (WS 269–2019), following the principles of early, combined, and sufficient antimicrobial therapy (Liu et al., 2022).

### 2.4 Model construction, evaluation and validation

Feature selection was performed in two stages. Initially, the Boruta algorithm, based on a random forest classifier, was applied to identify all-relevant features by comparing their importance scores with randomized shadow attributes. Subsequently, recursive feature elimination (RFE), also based on a random forest estimator, was used to refine feature selection and identify the optimal subset of variables that contributed most significantly to classification performance. Feature selection was nested within each cross-validation split to avoid information leakage. Performance curves indicated that predictive ability plateaued after the inclusion of eight variables; therefore, the top eight predictors were retained for model construction.

The dataset was randomly split into a training set (70%) and a test set (30%) using a fixed random seed to ensure reproducibility. Using Python-based libraries such as scikit-learn and XGBoost, six supervised machine learning algorithms were constructed: support vector machine (SVM), extreme gradient boosting (XGBoost), light gradient boosting machine (LightGBM), random forest (RF), multilayer perceptron (MLP), and logistic regression (LR). All six algorithms were trained using these 8 features. Hyperparameters for each algorithm were optimized through grid search in combination with five-fold cross-validation to ensure robustness and avoid overfitting. In addition, tree-based models were constrained by limiting maximum tree depth, and both tree-based and linear models incorporated regularization techniques to further reduce overfitting and enhance generalizability.

To address the class imbalance between recovery and chronic cases, the Synthetic Minority Oversampling Technique (SMOTE) was applied to the training dataset. This method generates synthetic samples of the minority class based on feature-space similarities between existing minority instances, thereby improving representation without duplicating records. The SMOTE procedure was performed only within the training set to avoid data leakage, and models trained on both original and SMOTE-balanced data were compared for robustness.

Model performance was evaluated using multiple metrics, including the area under the receiver operating characteristic curve (AUC), calibration plots, and decision curve analysis (DCA). These evaluations were conducted on both the training and testing sets to assess discrimination, calibration, and clinical utility.

To enhance interpretability, SHapley Additive exPlanations (SHAP) were used to quantify the contribution of each input feature to model predictions. SHAP-based visualizations, including summary plots, dependence plots, and beeswarm diagrams, were generated to demonstrate the influence of individual variables on chronic brucellosis risk. Higher SHAP values indicated stronger positive contributions to the model’s predicted probability, while negative SHAP values suggested suppressive effects.

Finally, an interactive web-based prediction tool was developed using the Streamlit framework. The tool enables real-time input of clinical variables and visual feedback via single-sample SHAP force plots, making the model accessible and interpretable for clinical users and researchers alike.

### 2.5 Statistical analysis

All statistical analyses were conducted using R software (version 4.3.0) and Python (version 3.10.6). Missing values were addressed through multiple imputation with the “mice” package, and variables with more than 20% missingness had already been excluded during data collection. Continuous variables conforming to a normal distribution were presented as mean ± standard deviation (SD), and intergroup comparisons were assessed using independent-sample t tests. For continuous variables that did not follow a normal distribution, results were expressed as median and interquartile range [M (Q_1_, Q_3_)], with differences evaluated via the Mann–Whitney U test. Categorical data were summarized as frequencies and proportions [n (%)], and analyzed using the chi-square test or Fisher’s exact test, depending on sample size. A p-value < 0.05 (two-tailed) was considered indicative of statistical significance.

## 3 Results

The baseline clinical characteristics of the enrolled patients are summarized in **Table 2**. A total of 555 patients were included in the study, of whom 144 (25.95%) progressed to chronic brucellosis (chronic group), while 411 (74.05%) recovered without chronicity (recovery group). Compared to the recovery group, patients in the chronic group exhibited significantly higher incidences of arthralgia, myalgia, and arthritis, while reporting lower rates of fever, headache, and splenomegaly (all p < 0.05). These findings suggest that specific symptom clusters and organ involvement patterns may serve as early indicators of chronic disease progression.

**Table 2:** Baseline clinical characteristics of patients with and without chronic brucellosis.

Variables	Total (n = 555)	Recovery group (n = 411)	Chronic group (n = 144)	P
Demographics				
Age	52.3 ± 14.3	51.8 ± 15.0	53.8 ± 11.9	0.131
Gender				0.239
Female	160 (28.83)	124 (30.17)	36 (25.00)	
Male	395 (71.17)	287 (69.83)	108 (75.00)	
Symptoms				
Fever	438 (78.92)	351 (85.40)	87 (60.42)	< 0.001
Myalgia	246 (44.32)	172 (41.85)	74 (51.39)	0.047
Fatigue	376 (67.75)	279 (67.88)	97 (67.36)	0.908
Anorexia	337 (60.72)	255 (62.04)	82 (56.94)	0.281
Headache	100 (18.02)	87 (21.17)	13 (9.03)	0.001
Arthralgia				< 0.001
No arthralgia	276 (49.73)	238 (57.91)	38 (26.39)	
Monoarticular pain	182 (32.79)	116 (28.22)	66 (45.83)	
Polyarticular pain	97 (17.48)	57 (13.87)	40 (27.78)	
Organ Involvement				
Hepatomegaly	62 (11.17)	51 (12.41)	11 (7.64)	0.118
Splenomegaly	253 (45.59)	205 (49.88)	48 (33.33)	< 0.001
Arthritis	171 (30.81)	102 (24.82)	69 (47.92)	< 0.001
Neurobrucellosis	27 (4.86)	19 (4.62)	8 (5.56)	0.654
Cardiac involvement in brucellosis	7 (1.26)	4 (0.97)	3 (2.08)	0.383
Genitourinary involvement in brucellosis	34 (6.13)	24 (5.84)	10 (6.94)	0.634

Laboratory findings revealed multiple statistically significant differences between the recovery and chronic groups as shown in **Table 3**. The chronic group had higher PLT, eosinophils, ALB, PA, BUN, UA, Cl, TC and HDL-C, and lower ALT, AST, GLB, GGT, PT, APTT, D-D, FIB-C, TG, ESR, PCT, and CCP. Positive blood culture was significantly less frequent in the chronic group. These alterations suggest significant involvement of hepatic function, coagulation pathways, lipid metabolism, and systemic inflammation in the pathophysiological transition toward chronic brucellosis.

**Table 3:** Laboratory findings of patients with and without chronic brucellosis.

Variables	Total (n = 555)	Recovery group (n = 411)	Chronic group (n = 144)	P
Complete Blood Count				
WBC, 10^^9^ /L	4.90 (3.70, 6.60)	4.90 (3.60, 6.70)	4.90 (4.00, 6.53)	0.987
RBC, 10^^12^ /L	4.17 (3.76, 4.53)	4.13 (3.74, 4.49)	4.24 (3.84, 4.65)	0.116
HGB, g/L	124.00 (111.00, 137.00)	124.00 (111.00, 135.00)	126.50 (113.00, 142.25)	0.989
PLT, 10^^9^ /L	206.00 (140.00, 267.00)	197.00 (138.50, 261.00)	220.00 (157.00, 279.00)	0.044
Lymphocytes, 10^^9^ /L	1.56 (1.20, 2.08)	1.56 (1.18, 2.08)	1.56 (1.29, 2.04)	0.974
Neutrophils, 10^^9^ /L	2.69 (1.77, 3.88)	2.64 (1.73, 4.04)	2.80 (2.07, 3.75)	0.267
Monocytes, 10^^9^ /L	0.41 (0.30, 0.59)	0.42 (0.30, 0.60)	0.40 (0.29, 0.55)	0.718
Eosinophil, 10^^9^ /L	0.03 (0.01, 0.09)	0.02 (0.01, 0.08)	0.06 (0.02, 0.15)	< 0.001
Basophil, 10^^9^ /L	0.01 (0.01, 0.02)	0.01 (0.01, 0.02)	0.02 (0.01, 0.02)	0.249
HCT, %	37.52 ± 5.44	37.27 ± 5.19	38.24 ± 6.05	0.065
MCV, fL	91.00 (87.35, 94.85)	90.90 (87.30, 94.50)	91.15 (87.40, 94.90)	0.73
MCH, pg	30.00 (28.65, 31.35)	30.00 (28.65, 31.30)	30.10 (28.67, 31.63)	0.072
MCHC, g/L	329.00 (322.00, 337.00)	329.00 (321.50, 337.00)	328.00 (322.00, 337.00)	0.569
RDW-CV, %	13.50 (12.80, 14.50)	13.50 (12.80, 14.35)	13.50 (12.80, 14.72)	0.132
RDW-SD, fL	44.00 (41.70, 47.30)	43.80 (41.75, 47.05)	44.65 (41.70, 48.23)	0.049
PDW, fL	15.30 (11.80, 16.30)	15.40 (11.70, 16.30)	15.15 (11.90, 16.10)	0.646
P-LCR, %	25.50 (17.90, 32.60)	25.70 (18.20, 32.50)	24.90 (16.90, 33.73)	0.951
PCT-PLT, %	0.20 (0.15, 0.26)	0.20 (0.14, 0.25)	0.21 (0.16, 0.26)	0.084
MPV, fL	10.10 (9.00, 11.00)	10.10 (9.00, 10.90)	10.00 (8.70, 11.10)	0.993
Liver Function Tests				
ALT, U/L	33.00 (19.00, 61.00)	39.00 (22.00, 70.00)	23.00 (15.00, 38.25)	< 0.001
AST, U/L	32.00 (21.00, 53.00)	35.00 (23.00, 59.00)	25.00 (18.00, 35.25)	< 0.001
TP, g/L	63.40 (59.00, 67.40)	63.20 (59.00, 67.40)	63.55 (59.08, 67.28)	0.812
ALB, g/L	35.18 ± 5.04	34.69 ± 4.84	36.58 ± 5.32	< 0.001
GLB, g/L	27.20 (24.45, 31.10)	27.70 (25.00, 31.30)	26.05 (23.10, 29.27)	0.002
PA, mg/L	158.00 (115.00, 209.50)	146.00 (113.50, 200.50)	183.00 (121.00, 237.50)	< 0.001
TBIL, μmol/L	11.30 (8.35, 15.50)	11.40 (8.40, 15.50)	11.05 (8.25, 15.43)	0.628
DBIL, μmol/L	4.40 (3.00, 6.10)	4.50 (3.00, 6.20)	4.00 (3.00, 5.90)	0.537
IBIL, μmol/L	6.70 (5.10, 9.70)	6.70 (5.20, 9.60)	6.70 (5.00, 9.93)	0.83
ALP, U/L	94.00 (73.00, 126.00)	96.00 (74.00, 132.50)	89.00 (69.75, 111.00)	0.075
GGT, U/L	48.00 (27.00, 104.00)	53.00 (29.00, 116.00)	35.50 (22.00, 75.00)	< 0.001
TBA, μmol/L	4.30 (2.90, 7.80)	4.30 (2.90, 7.75)	4.90 (2.88, 8.50)	0.537
Renal Function Tests& Electrolytes				
BUN, mmol/L	4.05 (3.19, 5.41)	3.85 (3.05, 5.13)	4.71 (3.69, 5.99)	< 0.001
CRE, μmol/L	60.00 (52.00, 70.00)	59.70 (52.00, 70.00)	60.00 (51.00, 71.47)	0.793
UA, μmol/L	237.00 (181.50, 296.00)	227.00 (172.50, 288.00)	269.00 (212.75, 319.50)	< 0.001
K, mmol/L	3.95 (3.70, 4.22)	3.93 (3.67, 4.21)	3.99 (3.74, 4.31)	0.101
Na, mmol/L	138.00 (135.00, 140.00)	138.00 (135.00, 140.00)	139.00 (137.00, 140.25)	0.776
Cl, mmol/L	103.10 (100.00, 105.40)	103.00 (99.80, 105.25)	103.40 (100.97, 105.82)	0.029
Coagulation Function Tests				
PT, s	12.90 (12.10, 13.80)	13.10 (12.20, 13.90)	12.65 (11.90, 13.60)	0.022
PT-S, %	81.40 (71.05, 92.00)	80.20 (70.70, 90.90)	82.60 (72.35, 96.50)	0.074
INR	1.11 (1.04, 1.21)	1.12 (1.04, 1.21)	1.08 (1.03, 1.17)	0.197
APTT, s	32.30 (28.90, 36.75)	33.00 (29.30, 37.60)	30.05 (27.60, 33.85)	< 0.001
TT, s	17.00 (16.10, 17.90)	16.90 (15.90, 17.80)	17.10 (16.30, 18.40)	0.209
D-D, mg/L	1.33 (0.61, 4.40)	1.59 (0.70, 5.68)	0.91 (0.38, 2.54)	< 0.001
FIB-C, g/L	3.47 (2.71, 4.75)	3.59 (2.79, 4.78)	3.21 (2.51, 4.42)	0.003
Blood Lipid Profile				
TC, mmol/L	3.70 (3.22, 4.40)	3.69 (3.21, 4.33)	3.88 (3.34, 4.78)	0.008
TG, mmol/L	1.35 (0.95, 1.91)	1.43 (1.04, 1.97)	1.09 (0.85, 1.60)	< 0.001
HDL-C, mmol/L	0.84 (0.69, 1.04)	0.80 (0.68, 0.97)	0.97 (0.80, 1.21)	< 0.001
LDL-C, mmol/L	2.19 (1.80, 2.67)	2.16 (1.77, 2.64)	2.29 (1.89, 2.78)	0.227
Inflammatory Markers				
ESR, mm/h	25.00 (12.00, 50.00)	29.00 (15.00, 50.00)	20.00 (10.00, 45.00)	0.008
PCT, ng/mL	0.14 (0.06, 0.31)	0.16 (0.07, 0.33)	0.08 (0.05, 0.20)	< 0.001
CCP, RU/ml	12.40 (10.10, 16.05)	13.00 (10.10, 17.00)	11.65 (9.70, 14.03)	< 0.001
CRP, mg/L				< 0.001
≤6	335 (60.36)	227 (55.23)	108 (75.00)	
>6	220 (39.64)	184 (44.77)	36 (25.00)	
Diagnostic Tests				
Blood culture positive	117 (21.08)	105 (25.55)	12 (8.33)	< 0.001
SAT				0.313
1:25	16 (2.88)	12 (2.92)	4 (2.78)	
1:50	56 (10.09)	38 (9.25)	18 (12.5)	
1:100	79 (14.23)	52 (12.65)	27 (18.75)	
1:200	305 (54.95)	236 (57.42)	69 (47.92)	
1:400	26 (4.68)	19 (4.62)	7 (4.86)	
not performed	73 (13.15)	54 (13.14)	19 (13.19)	

**Figure 1:**
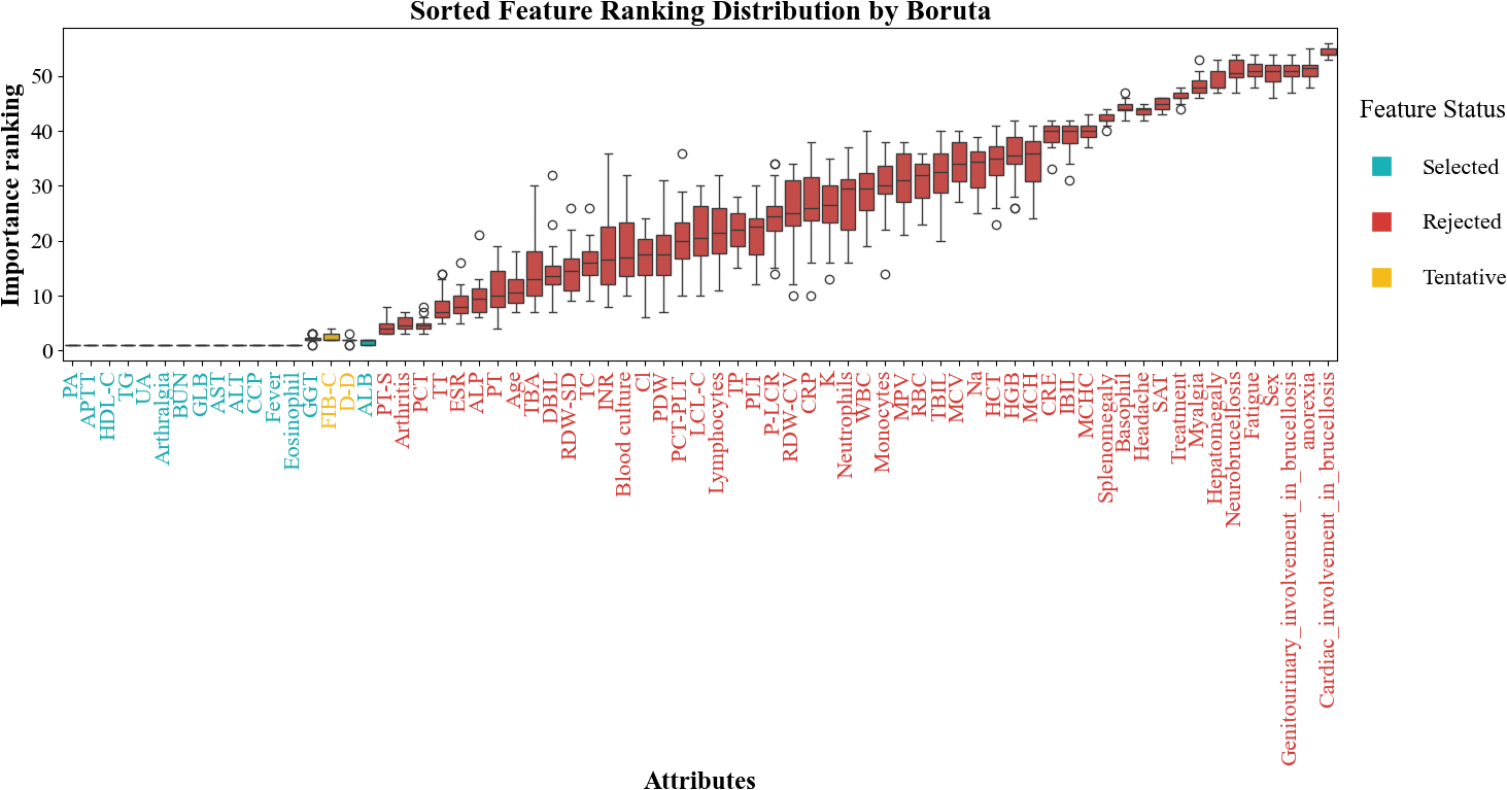
Feature selection results based on Boruta algorithm.

To identify the most informative predictive variables, we applied the Boruta algorithm for feature selection. As illustrated in **Figure 1**, a total of 14 variables were deemed important (colored in cyan), including BUN, ALT, GLB, PA, UA, TG, HDL-C, AST, eosinophil, APTT, CCP, arthralgia, fever, and ALB. These features demonstrated significantly higher importance scores than rejected or tentative variables.

**Figure 2:**
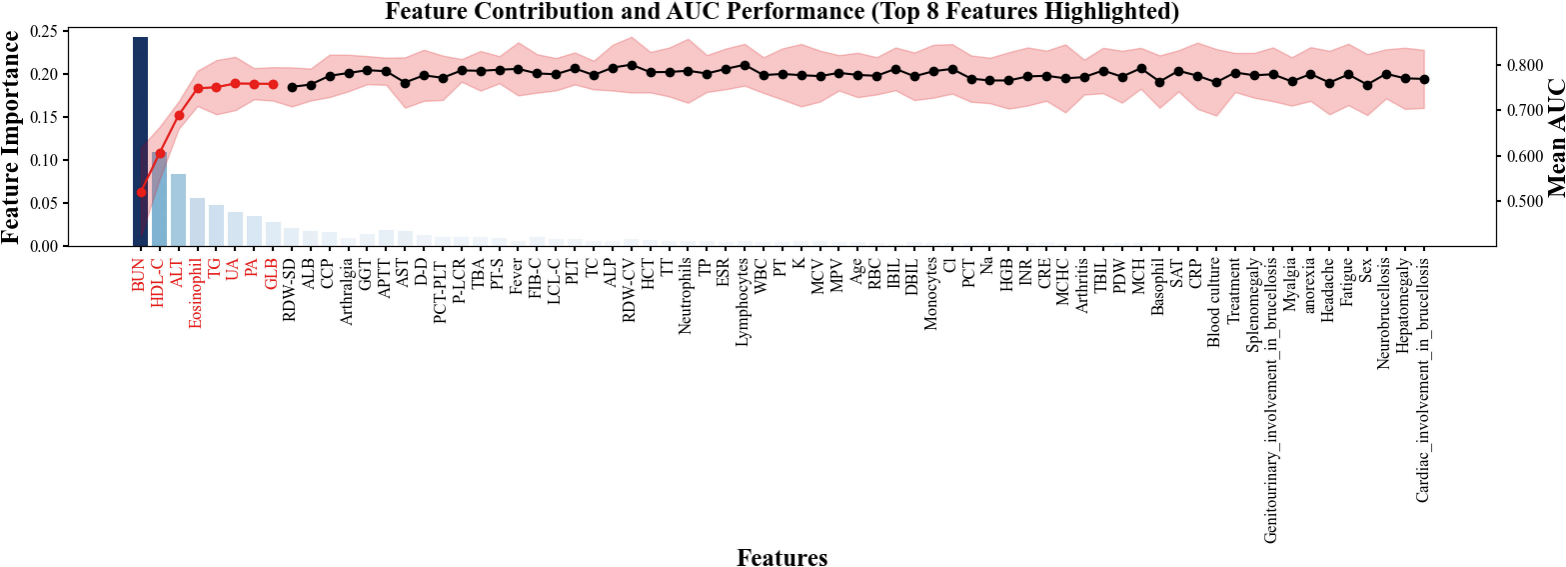
Feature contribution and model performance in sequential feature.

Subsequently, the top-ranking features were incorporated sequentially to evaluate their cumulative impact on model performance. As shown in **Figure 2**, model performance improved rapidly with the initial features and plateaued after the top 8 were included, suggesting that most of the predictive power was concentrated within this subset. Therefore, the top 8 features—BUN, HDL-C, ALT, eosinophil, TG, UA, PA, and GLB—were selected for final model construction.

**Figure 3:**
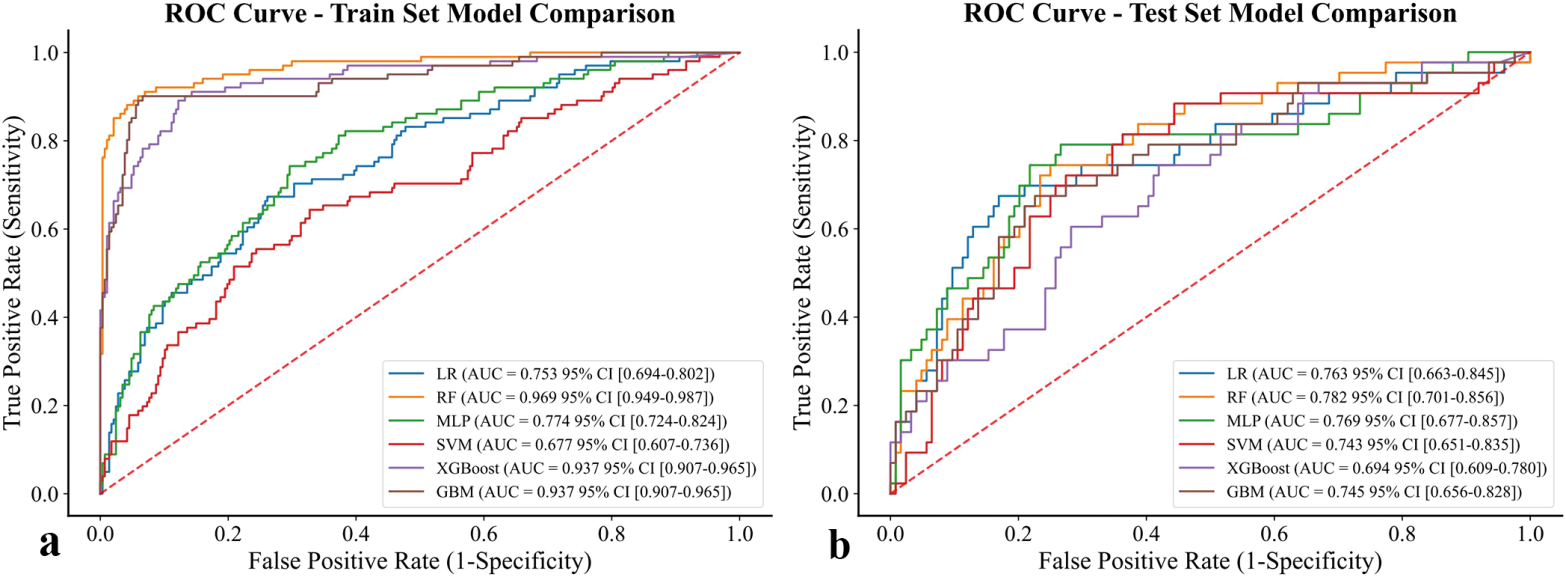
ROC curve of six models for prediction of chronic brucellosis. **(a)** training set **(b)** test set.

The performance of six supervised machine learning models was compared using ROC curves, as shown in **Figure 3**. In the training set, ensemble methods including RF, XGBoost, and LightGBM exhibited excellent discrimination with AUC values above 0.93, while LR (AUC = 0.753), SVM (AUC = 0.677), and MLP (AUC = 0.774) demonstrated lower predictive ability, suggesting that the tree-based algorithms captured the underlying patterns more effectively. In the test set, however, performance decreased across all models, reflecting reduced generalizability. RF achieved the highest AUC of 0.782 (95% CI: 0.701 - 0.856), followed closely by MLP (0.769) and LR (0.763). In contrast, LightGBM showed the lowest discrimination (AUC = 0.745), and SVM remained relatively weak (AUC = 0.754). Taken together, these results indicate that although tree-based methods dominated in the training set, RF and MLP showed relatively better robustness in the test set, highlighting their potential suitability for predicting chronic brucellosis in independent cohorts.

To further assess the influence of class imbalance, additional analyses were conducted after applying the SMOTE to the training data (**Supplementary Figure 1**). Following oversampling, AUC and F1-scores of some algorithms (particularly LR and SVM) increased modestly, whereas ensemble models such as RF remained highly stable, demonstrating consistent performance across both the original and balanced datasets. These findings support the robustness of the RF model and indicate that the observed superiority of tree-based methods was not driven by data imbalance.

**Figure 4:**
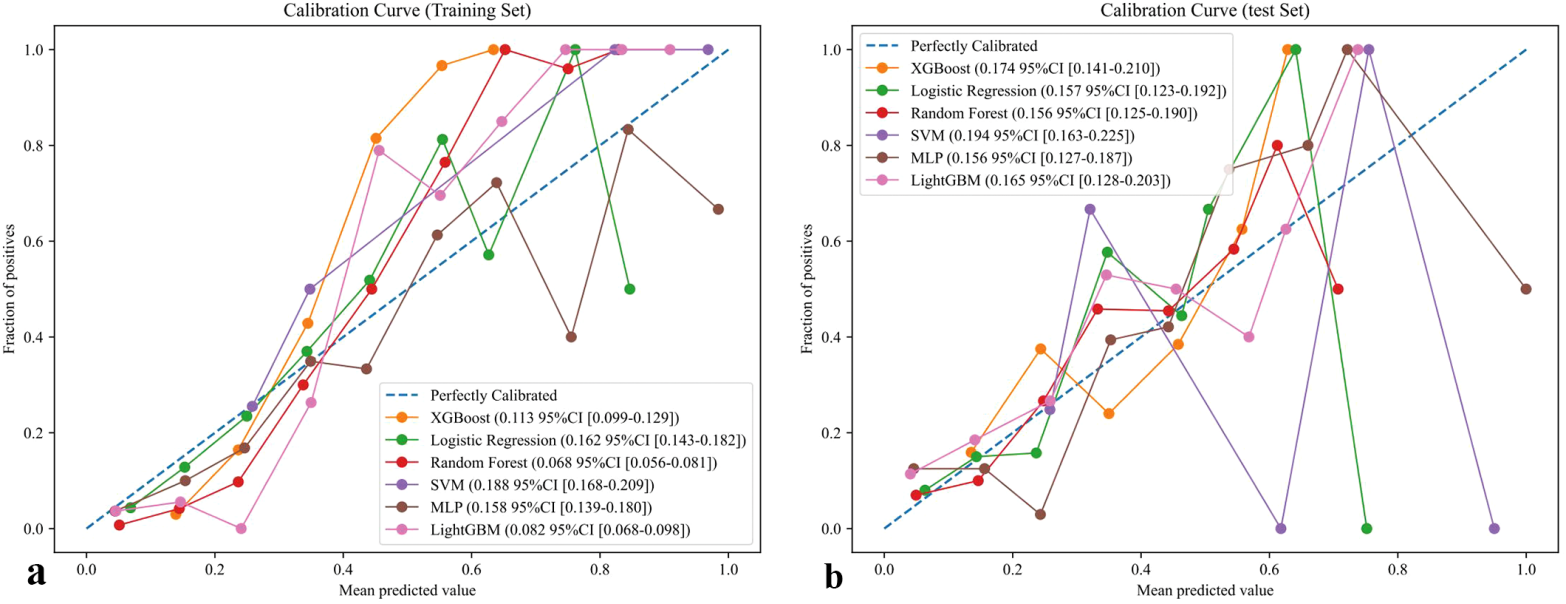
Calibration curve of six models for prediction of chronic brucellosis. **(a)** training set **(b)** test set.

Calibration analysis is shown in **Figure 4**. In the training set, RF achieved the best calibration performance (Emax = 0.058, 95% CI: 0.056 - 0.081), followed by LightGBM (Emax = 0.082) and XGBoost (Emax = 0.113). In contrast, SVM and MLP exhibited substantial deviation from the ideal calibration line, reflecting poor probability estimation. Similar results were observed in the test set, where RF again demonstrated the most favorable agreement between predicted and observed risks (Emax = 0.155, 95% CI: 0.123 - 0.187), outperforming LightGBM (0.165) and XGBoost (0.174). These findings suggest that RF provided the most reliable probability estimates across both datasets. To further evaluate the effect of class imbalance on model calibration, additional analyses were performed after applying SMOTE to the training data (**Supplementary Figure 2**). The overall calibration trends remained consistent with the primary results, with RF maintaining the most stable and well-calibrated probability predictions among all algorithms.

**Figure 5:**
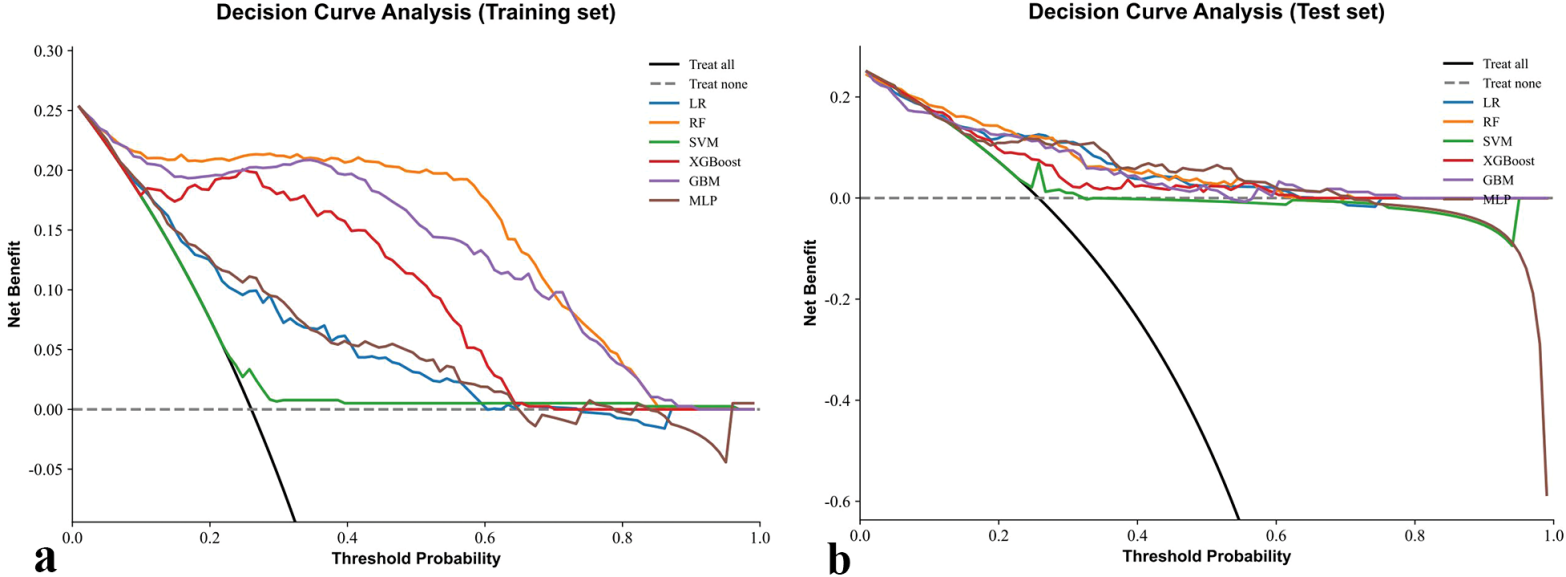
DCA of six models for prediction of chronic brucellosis. **(a)** training set **(b)** test set.

Decision curve analysis results are presented in **Figure 5**. In the training set, RF consistently provided the highest net benefit across a wide range of threshold probabilities, indicating superior clinical utility. XGBoost and LightGBM also showed favorable performance but were consistently outperformed by RF, whereas SVM and MLP offered little to no net clinical benefit. In the test set, RF again yielded the greatest net benefit, confirming its robustness and practical value for clinical application. To verify the stability of clinical utility under class imbalance adjustment, we additionally performed DCA after applying SMOTE to the training data (**Supplementary Figure 3**). The overall net benefit profiles remained comparable to the primary analysis, with RF maintaining the broadest range of positive net benefit across threshold probabilities. Minor changes in the magnitude of net benefit were observed for other algorithms, but the ranking order and clinical interpretation were largely unchanged.

Predictive performance metrics on the test set are presented in **Table 4**. Overall, RF demonstrated the most stable and balanced performance across multiple evaluation indices. RF achieved an accuracy of 0.76, comparable to LR and LightGBM, and achieved relatively higher sensitivity than XGBoost and SVM; however, the absolute sensitivity value remained modest, underscoring the need for further optimization. Importantly, RF maintained competitive F1 and kappa scores, reflecting a strong balance between precision and recall as well as agreement with ground truth labels. By contrast, SVM consistently showed the weakest performance across all metrics. Taken together, RF exhibited the most balanced performance across metrics, suggesting potential clinical applicability for predicting chronic brucellosis.

**Table 4:** Comparison of predictive metrics for six models on the test set.

Metrics	SVM	XGBoost	LightGBM	Logistic regression	Random forest	MLP
accuracy	0.73	0.77	0.76	0.77	0.76	0.79
sensitivity	0.00	0.18	0.30	0.23	0.30	0.25
specificity	0.99	0.97	0.92	0.95	0.91	0.97
F1 score	0.00	0.29	0.40	0.34	0.39	0.38
Kappa score	-0.01	0.21	0.27	0.24	0.26	0.29

Among the compared algorithms, RF performed best overall, with moderate discrimination and well-calibrated probabilities but limited sensitivity; therefore, its use should be regarded as exploratory and intended to assist rather than replace clinical judgment.

**Figure 6:**
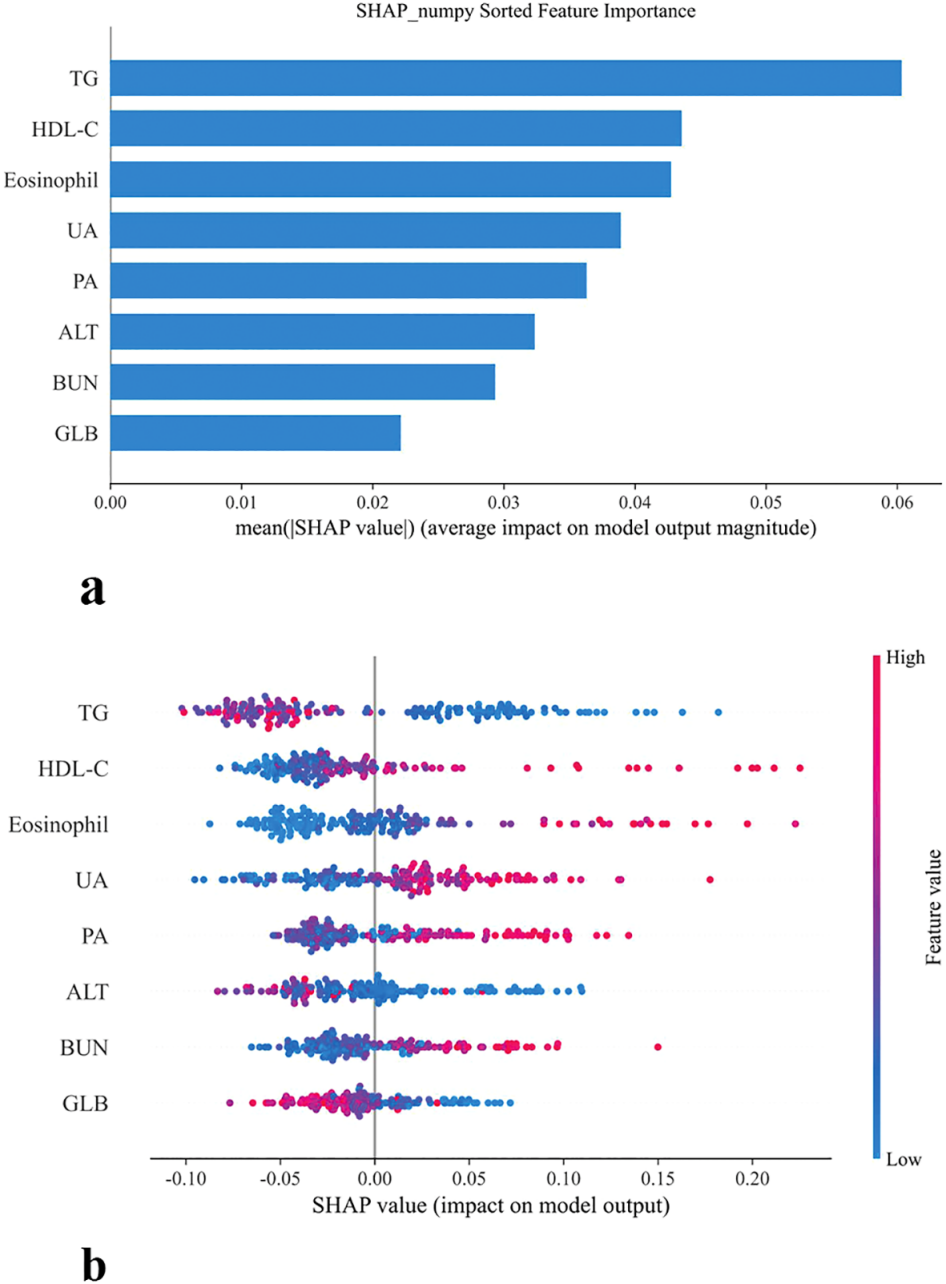
SHAP-based feature importance and distribution in model prediction **(a)** Bar plot of mean SHAP values **(b)** SHAP summary plot.

Feature importance analysis based on the RF model is presented in **Figure 6**. Panel (a) ranks predictors according to their mean absolute SHAP values, with TG emerging as the most influential feature, followed by HDL-C, eosinophil count, UA, PA, ALT, BUN, and GLB. These findings highlight that both lipid metabolism indicators (TG, HDL-C) and immune-inflammatory markers (eosinophils, GLB) play central roles in RF-driven risk stratification for chronic brucellosis.

The SHAP summary plot further illustrates the directional impact of individual variables on the RF model’s output. Higher levels of HDL-C, eosinophils, UA, PA, and BUN were associated with positive SHAP values, indicating an increased probability of chronic disease. Conversely, TG, GLB, and ALT exerted negative SHAP contributions, and the higher values suggested potential protective effects. Importantly, these patterns are consistent with the clinical relevance of lipid and immune dysregulation in chronic infection, underscoring the robustness of the RF model in capturing biologically meaningful predictors.

To further examine the stability of feature importance under class imbalance adjustment, SHAP analysis was repeated after applying SMOTE to the training data (**Supplementary Figure 4**). The same eight features were consistently identified as the top predictors, with only minor shifts in their relative ranking. This high degree of overlap indicates that the feature–outcome associations captured by the RF model remained stable despite resampling, confirming the intrinsic robustness and biological relevance of the identified predictors.

**Figure 7:**
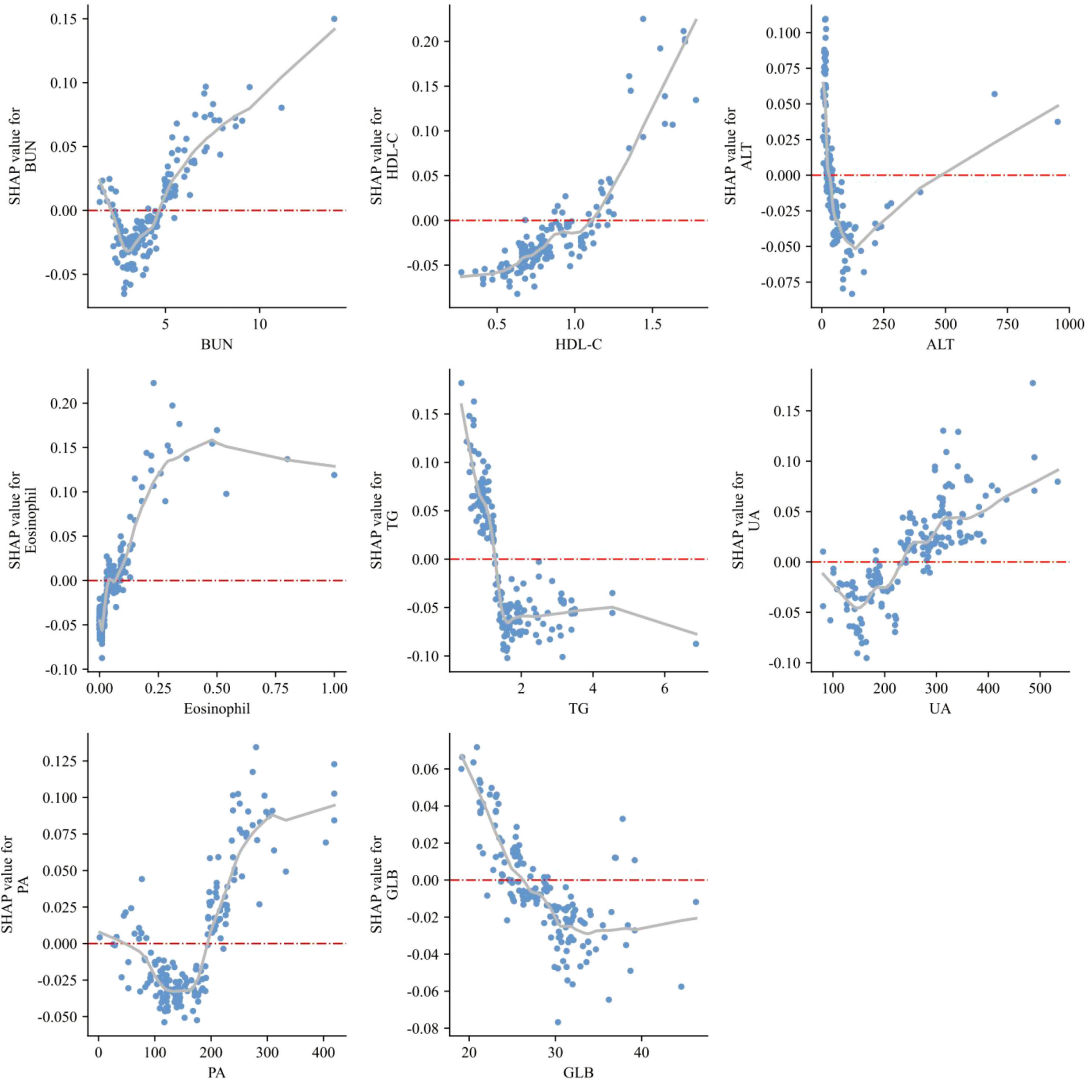
SHAP dependence plots of top predictive features.

**Figure 7** displays SHAP dependence plots for the eight most influential variables identified by the RF model, illustrating their nonlinear effects on prediction outcomes. BUN, HDL-C, eosinophil count, and UA showed positive associations with risk, where higher values corresponded to increased SHAP values and thus greater chronicity probability. In contrast, TG and GLB exhibited inverse associations, with lower levels driving elevated risk, and ALT demonstrated a pronounced negative relationship as well, with reduced levels strongly linked to higher predicted probability. PA displayed a J-shaped curve, indicating that both very low and high concentrations may contribute to chronic progression.

These dependence plots highlight the heterogeneous influence and threshold effects of biochemical and immune-inflammatory indicators, reinforcing their biological plausibility. By revealing feature-specific inflection points, the RF-based SHAP analysis enhances model transparency and supports its clinical interpretability in the prediction of chronic brucellosis.

**Figure 8:**
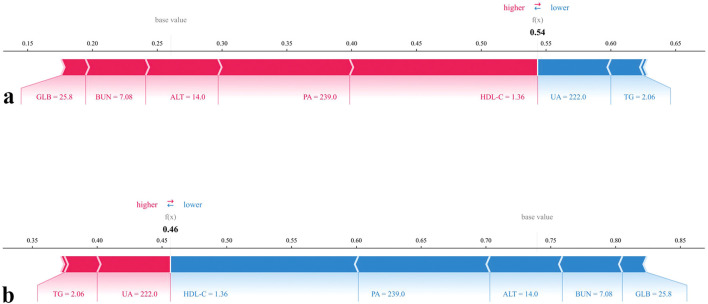
SHAP force plots for individual predictions by the chronic brucellosis model. **(a)** SHAP force plot for the prediction of having Chronic progression. **(b)** SHAP force plot for the prediction of not having Chronic progression.

In addition, two case-level force plots were provided to illustrate the interpretability of the model (**Figure 8**). In these plots, red features indicate positive contributions to the prediction (increasing risk), whereas blue features indicate negative contributions (decreasing risk). The driving factors varied across individuals: in case (a), higher GLB (25.8), BUN (7.08), ALT (14.0), PA (239.0), and HDL-C (1.36) collectively increased the predicted probability of chronic brucellosis, whereas lower UA (222.0) and TG (2.06), provided modest protective effects. Conversely, in case (b), elevated TG (2.06) and UA (222.0) acted as risk-enhancing contributors, while higher HDL-C (1.36), PA (239.0), ALT (14.0), BUN (7.08), and GLB (25.8) exerted strong protective influences.

Together, these SHAP-based interpretability tools provide robust insights into the contribution, directionality, and threshold behavior of individual predictors. By uncovering feature-specific inflection points, they enhance the clinical interpretability of the model and support its application in real-world decision-making.

**Figure 9:**
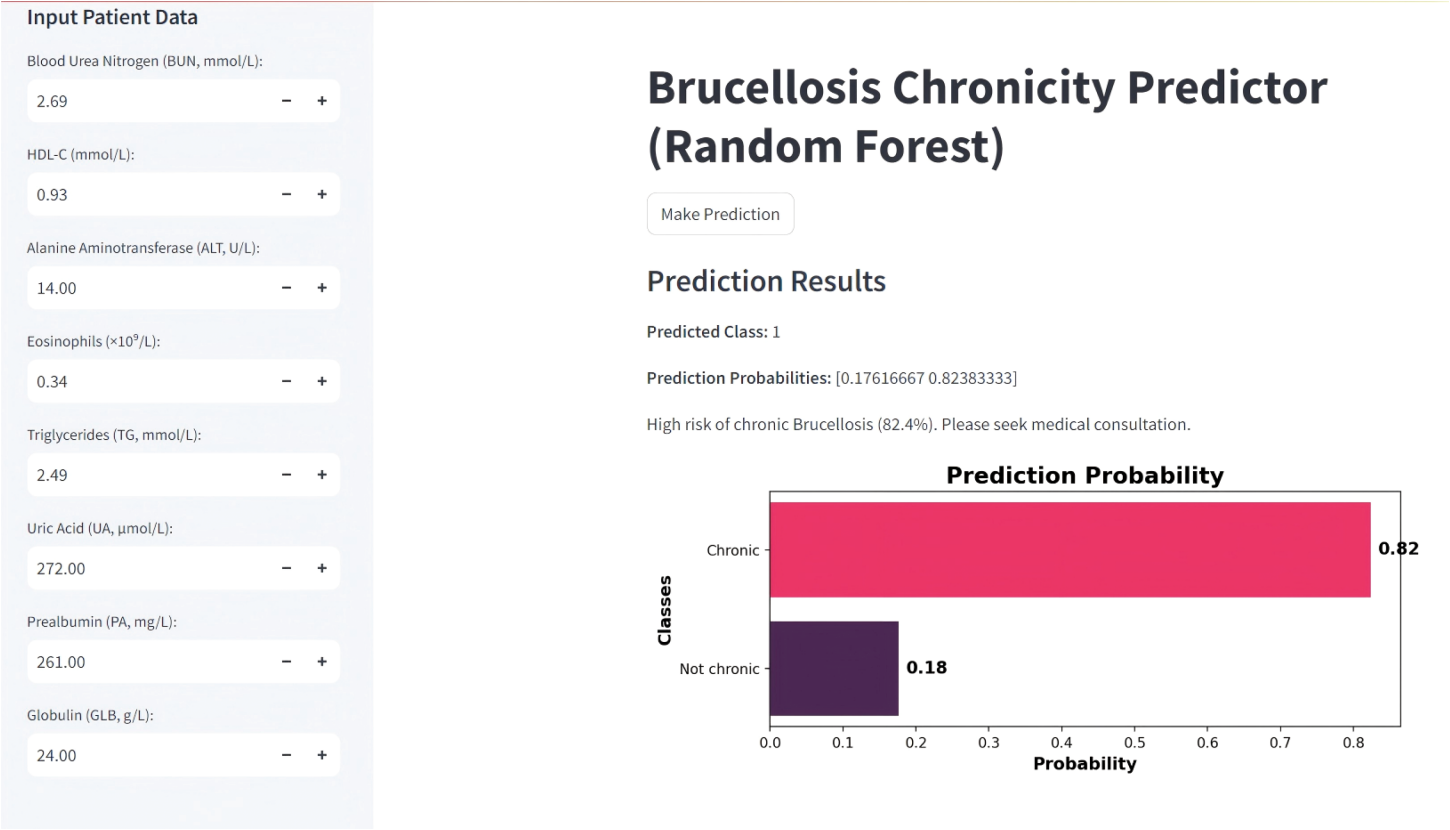
Web-based prediction interface of the Random Forest model for chronic brucellosis risk assessment.

To facilitate clinical application and enhance accessibility, we developed an online risk prediction tool based on the final model incorporating the top-ranking SHAP features. All selected variables are routinely available in clinical settings, allowing for convenient input and real-time prediction. As illustrated in **Figure 9**, ‘1’ indicates a positive prediction for chronic progression, while ‘0’ denotes a negative prediction. The value in parentheses represents the predicted probability. The web-based risk calculator is publicly available at: https://brucellosis-prediction-rf-hm4jkzjnytrnaygmhqvevk.streamlit.app/, offering clinicians an intuitive platform to assess individual patient risk profiles based on key clinical indicators. To ensure transparency and reproducibility, the full implementation code has also been made available at https://github.com/moresaying98/Brucellosis-Prediction-RF/blob/main/Firday.py.

## 4 Discussion

In this study, we developed and validated a machine learning-based model to predict chronic progression in patients with brucellosis, using a comprehensive set of clinical and laboratory features. Among six tested algorithms, RF achieved the best overall performance in terms of discrimination, calibration, and clinical utility. Its predictive power was further enhanced through SHAP, which enabled transparent interpretation of feature contributions.

Brucellosis remains a significant public health concern in endemic regions, owing to its zoonotic transmission, heterogeneous clinical manifestations, and substantial risk of chronic complications (Shen et al., 2022). In China alone, 684,380 cases were officially reported between 1950 and 2018, ranking brucellosis among the top ten notifiable infectious diseases nationwide (Yang et al., 2020). This sustained burden underscores the persistent challenges in interspecies transmission control and the unmet need for early identification of patients at risk of chronicity (Ghssein et al., 2025).

Although most cases of brucellosis are acute and responsive to treatment, a substantial proportion progress to chronic disease, resulting in increased morbidity and healthcare burden (Qureshi et al., 2023). Despite growing clinical awareness, reliable early prediction of chronicity remains elusive. Prior studies have predominantly focused on molecular and serological distinctions between acute and chronic stages, rather than on clinically applicable predictive modeling. For instance, differential expression of miR-1238-3p, miR-494, and miR-6069 has been proposed as potential markers of chronic disease (Budak et al., 2016), and proteomic analyses have identified several candidate proteins with predictive value for chronic progression (Li et al., 2024). However, these approaches are limited by high cost, limited accessibility, and lack of clinical validation. As a result, routinely available clinical and laboratory indicators remain the most feasible data sources for risk prediction in real-world practice.

Recently, ML has emerged as a powerful tool for disease forecasting and personalized risk assessment (Contreras and Vehi, 2018; Smith et al., 2023). ML applications in brucellosis have shown encouraging results in early detection, outbreak surveillance, and patient stratification. For example, Wang et al. developed a high-accuracy diagnostic model using support vector machines, although it did not address chronic progression (Wang et al., 2023). Shen et al. applied a convolutional long short-term memory (ConvLSTM) model to analyze the spatiotemporal dynamics of brucellosis in Europe, demonstrating superiority over conventional ARIMA approaches (Shen et al., 2022). Nonetheless, predictive modeling specifically targeting chronic brucellosis remains scarce. To our knowledge, the present study is the first to develop a clinically interpretable, ML-based risk prediction tool for chronic progression in brucellosis using routinely collected data.

In the final model, 8 variables were retained based on their contribution to predictive performance, including BUN, HDL-C, ALT, eosinophil, TG, UA, PA, and GLB. Although the directionality of some predictors may appear counterintuitive, this is consistent with the SHAP dependence plots. Such discrepancies between overall group differences and conditional model contributions likely reflect pathway interactions within tree-based algorithms.

Both TG and HDL-C emerged as critical lipid-related features in our model. Patients who progressed to chronic brucellosis tended to exhibit lower TG levels and higher HDL-C levels at admission - a pattern not previously reported in the brucellosis literature. While lipid metabolism dysregulation has been well-documented in various infectious and inflammatory conditions, including COVID-19 and HIV/AIDS (Funderburg and Mehta, 2016; Ryrsø et al., 2022). HDL-C is known for its pleiotropic immunomodulatory effects, including neutralization of lipopolysaccharide (LPS), attenuation of oxidative stress, and modulation of cytokine signaling (Bonacina et al., 2021). It has thus been hypothesized that elevated HDL-C may serve as a compensatory anti-inflammatory response in chronic infection. However, clinical evidence also points toward a U-shaped relationship between HDL-C and infection outcomes. In the ILLUMINATE trial, for example, pharmacologic elevation of HDL-C was paradoxically associated with increased infection-related mortality, despite improved lipid profiles (Barter et al., 2007). Mechanistically, HDL-C has been proposed to disrupt lipid rafts by depleting membrane cholesterol, potentially triggering unintended immune activation via protein kinase C signaling (van der Vorst et al., 2017). Therefore, the elevated HDL-C observed in chronic brucellosis may reflect either a protective adaptation or a maladaptive response contributing to persistent inflammation.

In contrast, lower TG levels were observed in chronic cases, opposing trends reported in diseases such as tuberculosis, where hypertriglyceridemia is linked to foam cell formation and impaired macrophage function (Agarwal et al., 2021). This discrepancy highlights pathogen-specific differences in host lipid reprogramming. One possible explanation is that Brucella infection induces an early hypometabolic shift or mitochondrial dysfunction, leading to TG depletion as part of a distinct metabolic phenotype. Altered hepatic lipid processing and systemic inflammation may further exacerbate this effect, potentially predisposing patients to chronic progression.

Eosinophils also emerged as a significant predictor of chronic brucellosis in our model, with higher counts observed in patients who progressed to chronic disease. This finding contrasts with most existing literature, which has primarily associated eosinopenia with brucellosis severity. For example, Jiao et al. reported that over 75% of patients exhibited eosinophil depletion at diagnosis, suggesting its value in early detection (Jiao et al., 2015). Similarly, Yang et al. found that eosinopenia correlated with higher complication rates and longer hospital stays (Yang et al., 2024). These studies indicate that eosinophil suppression may reflect systemic inflammatory burden in the acute phase.

However, several reports suggest that eosinophil levels may rise during recovery, and are often higher in chronic or prolonged cases (Jiang et al., 2017). This pattern aligns with our observations and supports the hypothesis that elevated eosinophil counts may reflect ongoing immune dysregulation or unresolved tissue injury in chronic disease states. In murine models of immune-mediated hepatic damage, eosinophils have been shown to infiltrate injured liver tissue and secrete interleukin-4, promoting hepatocyte proliferation and tissue regeneration (Aoki et al., 2021). While these data are primarily derived from tissue-level investigations, they suggest that peripheral eosinophil elevation in chronic brucellosis may serve as an indirect marker of localized immunologic remodeling or reparative activity. Nonetheless, the precise role of eosinophils in brucellosis pathogenesis - whether pathogenic, compensatory, or epiphenomenal - remains to be clarified. Further studies are warranted to determine whether eosinophilia in chronic brucellosis reflects a reactive immune phenotype, impaired resolution, or organ-specific immune responses not captured in peripheral blood.

In addition to conventional clinical and biochemical indicators, UA has been suggested as a potential predictor of chronicity. Although no prior studies have systematically examined the association between UA and chronic brucellosis, case reports have described patients with *Brucella*-induced septic arthritis who presented with hyperuricemia (Elzein and Sherbeeni, 2016). From a mechanistic perspective, brucellosis has been shown to impair both tubular and glomerular function, which may partially explain the elevated levels of UA observed in our chronic cohort (Conkar et al., 2018). Indeed, in our dataset, patients who progressed to chronic disease exhibited higher levels of BUN and CRE compared with acute cases, although these variables were not included in the final predictive model. This observation supports the hypothesis that increased UA may reflect renal dysfunction and could contribute to the risk of chronic progression.

Beyond brucellosis, elevated UA has been linked to adverse outcomes in other infectious and inflammatory conditions, reinforcing its potential role as a biomarker. A recent meta-analysis demonstrated that serum UA levels were significantly higher in patients with severe malaria compared to non-severe cases, and UA levels rose progressively with disease severity (Kuraeiad et al., 2023). Similarly, in chronic obstructive pulmonary disease (COPD), serum UA has been positively associated with acute exacerbations, and higher UA levels showed predictive value for AECOPD events (Zhao and Lv, 2024). Collectively, these findings suggest that UA may serve as a marker of systemic inflammation and metabolic-immune dysregulation across different disease contexts. At the pathophysiological level, UA, a byproduct of purine metabolism, can promote oxidative stress and low-grade systemic inflammation, which may provide a plausible explanation for its observed association with chronic infectious states (Qin et al., 2025).

PA was also identified as a relevant predictor, with higher PA values associated with an increased probability of chronic progression according to the SHAP analysis. Although low prealbumin is conventionally viewed as a marker of inflammation or malnutrition, PA levels may behave dynamically in the setting of chronic infection. In prolonged inflammatory states, the liver may respond to sustained immune activation with a compensatory upregulation of protein synthesis, resulting in relatively higher PA levels (Kaysen et al., 2002; Zinellu and Mangoni, 2021). This phenomenon has also been observed in other conditions, where PA reflects not only nutritional status but also hepatic synthetic response under persistent immune stress (Lim et al., 2005). In brucellosis, hepatic involvement and protein metabolic alterations are well documented, which may explain the positive association between PA and chronic progression observed in our model (Giambartolomei and Delpino, 2019).

Interestingly, the SHAP dependence pattern for ALT suggested an inverse relationship with chronic progression, where lower ALT values were associated with higher predicted chronic risk. Emerging evidence from other infectious settings suggests that low ALT levels may reflect hepatic immune suppression or metabolic dysfunction, rather than the absence of injury. For example, critically ill patients with reduced ALT have shown poorer outcomes, possibly due to impaired hepatocellular immunity or mitochondrial exhaustion (Itelman et al., 2022; Genzel et al., 2023). Similar findings in chronic hepatitis B indicate that ALT normalization may signal immune tolerance or insufficient cytotoxic response, rather than true resolution of inflammation (Jiang et al., 2023). In this context, our finding of low ALT being linked to chronic brucellosis risk may reflect a dysregulated hepatic immune response, potentially driven by Brucella's stealth mechanisms. Indeed, Brucella has been shown to induce a low-inflammatory, immune-tolerant environment within hepatic and reticuloendothelial tissues (Ahmed et al., 2016). These findings support the hypothesis that reduced ALT may serve as a surrogate marker of ineffective immune activation, although further validation in mechanistic studies is warranted.

BUN emerged as a significant variable in our model: higher baseline BUN was associated with greater risk of chronic progression. Although classically interpreted as a marker of reduced renal clearance or enhanced catabolism, BUN in infectious and inflammatory settings can also reflect broader systemic stress. Notably, in critically ill cohorts, elevated admission BUN independently predicts mortality even when serum creatinine is within the normal range, indicating prognostic information beyond overt renal failure (Beier et al., 2011). In brucellosis, host cells undergo metabolic reprogramming—including a Warburg-like shift and TCA-cycle attenuation—supporting the concept that nitrogen handling and organ-axis coordination may be perturbed during persistent infection (Czyż et al., 2017; Ponzilacqua-Silva et al., 2024). Taken together, higher BUN may serve as an accessible integrative marker of systemic metabolic stress rather than isolated renal impairment in patients at risk for chronic brucellosis, a hypothesis that warrants longitudinal and mechanistic validation.

GLB was identified as a key variable in our model, with lower values predicting higher chronic progression risk. As serum globulin integrates immunoglobulins, complement, and hepatic proteins, its decrease may reflect impaired humoral immunity or hepatic dysfunction. Persistent antigenic stimulation in chronic infections can induce T-cell exhaustion and immune dysregulation (Wherry, 2011). In *Brucella* infection, B-cell–T interactions have been shown to suppress CD4^+^ T-cell responses independent of antibody production, facilitating chronic persistence (Dadelahi et al., 2023). Hepatic involvement and disturbed protein metabolism may further reduce globulin synthesis (Giambartolomei and Delpino, 2019). Together, these findings suggest that decreased GLB may serve as an integrative marker of immune suppression and hepatic impairment in chronic brucellosis.

These mechanistic explanations are hypothesis-generating and require validation in prospective and mechanistic studies, as no direct evidence currently exists linking these biomarkers to chronic brucellosis. While our findings partially align with known patterns observed in other diseases, the pathophysiological implications of lipid and immune dysregulation in brucellosis require further investigation through mechanistic and longitudinal studies.

Although the RF model demonstrated the best overall discrimination and satisfactory calibration among the tested algorithms, its relatively low sensitivity limits its immediate clinical applicability. In real-world practice, this modest sensitivity indicates that some chronic brucellosis cases—particularly those at early or atypical stages—could be missed. Accordingly, the model should be viewed as a supplementary decision-support tool to assist clinicians in risk stratification rather than as an independent diagnostic method. Further optimization, including threshold fine-tuning, integration of additional biomarkers, and prospective external validation, will be essential to enhance recall and ensure safe, reliable translation into clinical practice.

This study has several notable strengths. First, it leverages a real-world clinical cohort from a brucellosis-endemic region, enhancing ecological validity. Second, the model’s interpretability via SHAP addresses a common limitation of machine learning in healthcare - namely, the lack of transparency in decision-making. Third, the deployment of the model as a web-based tool facilitates practical integration into clinical workflows and supports broader translational application.

This study has several limitations. First, the definition of chronic brucellosis is inherently heterogeneous across the literature and remains largely symptom-based; although our operational definition was guideline-consistent, the absence of universally accepted objective criteria may still introduce misclassification. Second, the retrospective, single-center design may have introduced selection bias, as only hospitalized patients were included. This design limits causal inference and underscores the need for cautious interpretation of associations identified by the model. Third, while the RF model outperformed other algorithms, its sensitivity in the test set remained limited, highlighting that the model should be regarded as exploratory and potentially used in combination with other clinical or molecular indicators. Forth, residual confounding cannot be excluded, as variables such as treatment adherence, initial regimen choice, and delay from symptom onset to therapy were not incorporated into the final model. Finally, the lack of external and temporal validation further restricts generalizability. External, multicenter, and prospective validation should be prioritized in future work to ensure the model’s stability and real-world applicability.

In conclusion, this study presents a clinically interpretable, machine learning-based model for early prediction of chronic brucellosis using routinely collected data. Our RF-based model shows promise as a clinically interpretable tool for early risk stratification. Nevertheless, external validation and integration with molecular markers are warranted before clinical adoption.

## Data Availability

The original contributions presented in the study are included in the article/**Supplementary Material**. Further inquiries can be directed to the corresponding author/s.
