# Supplementary figures and images for: Machine learning-based prediction model for chronic brucellosis: a multi-feature approach using clinical and laboratory data
Source: Front Cell Infect Microbiol. 2025 Nov 19;15:1700233. doi: 10.3389/fcimb.2025.1700233 (PMC12672903; doi:10.3389/fcimb.2025.1700233)

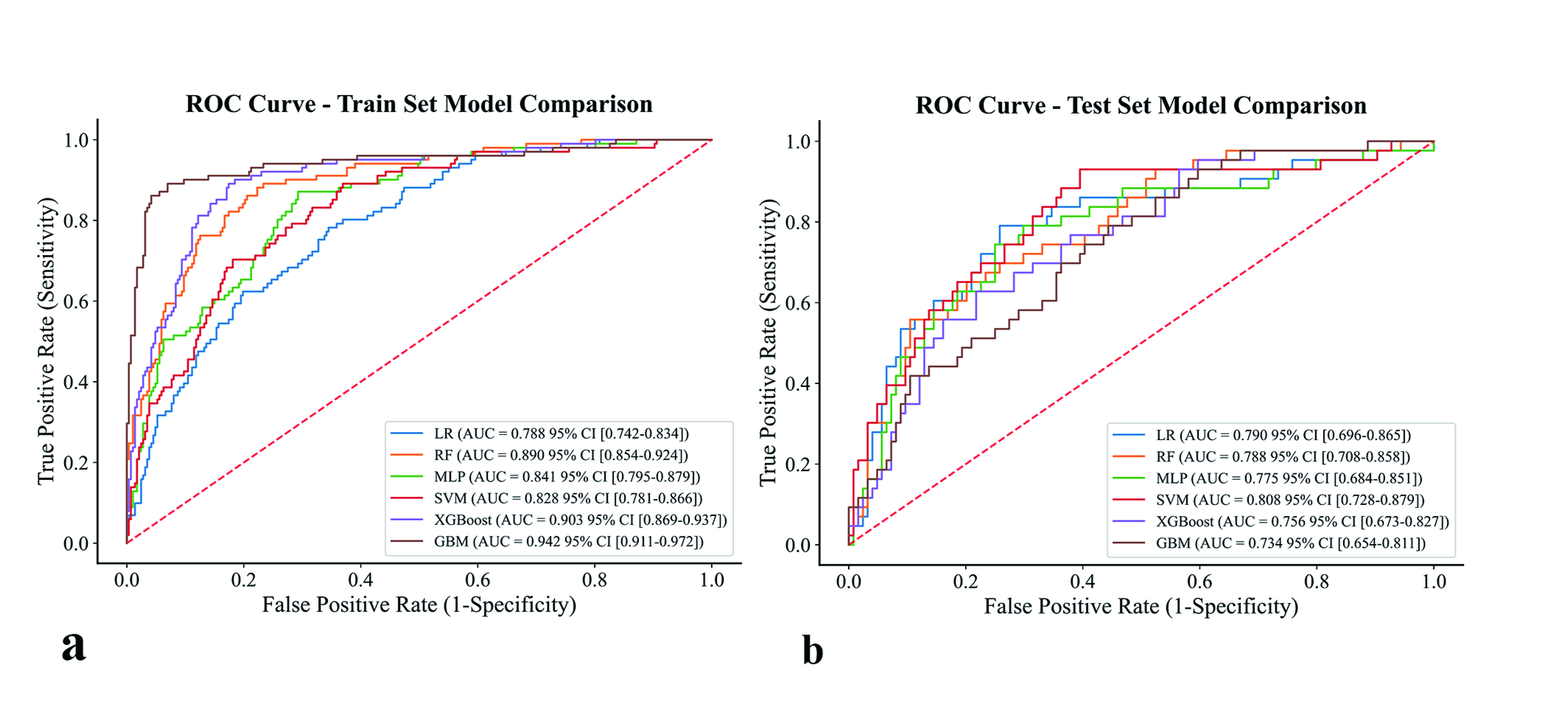

Supplement: Supplementary file 1 [file Image1.tif]

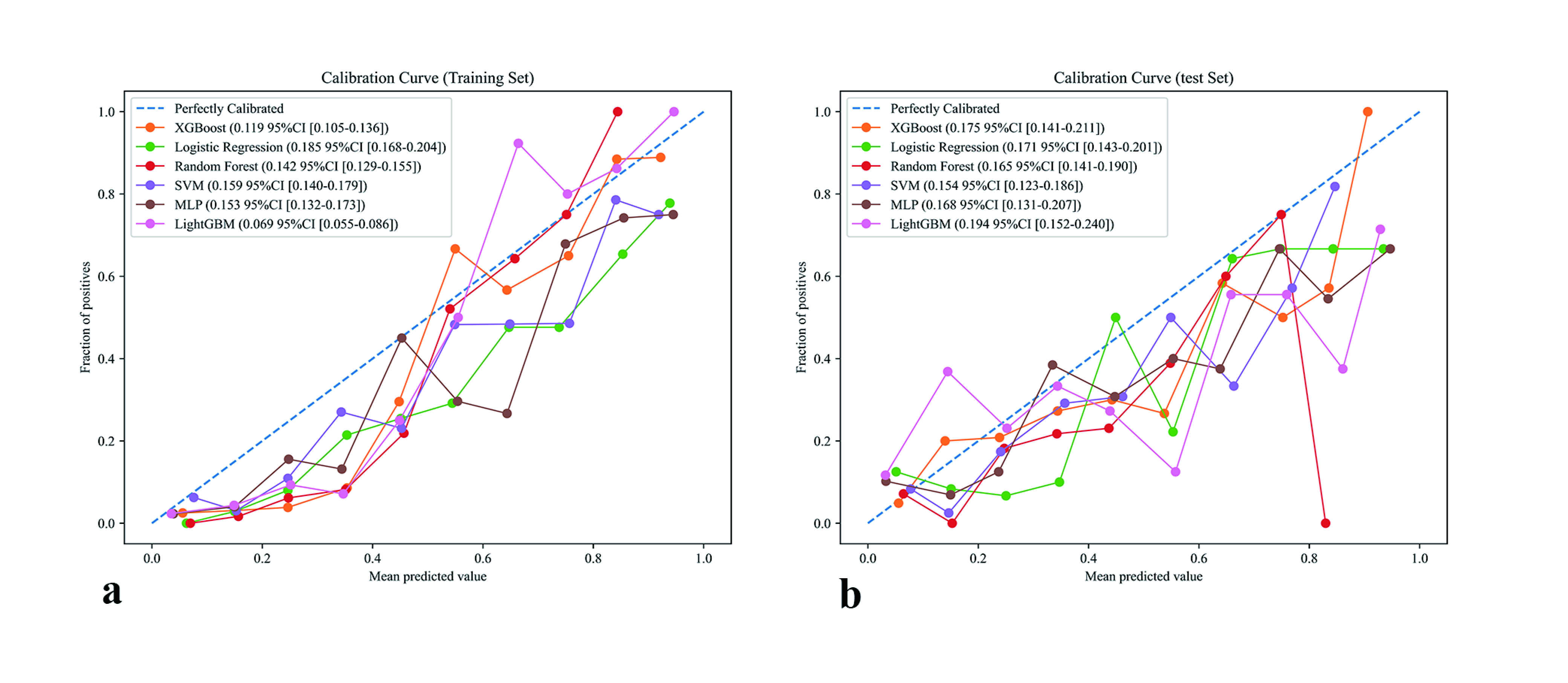

Supplement: Supplementary file 2 [file Image2.tif]

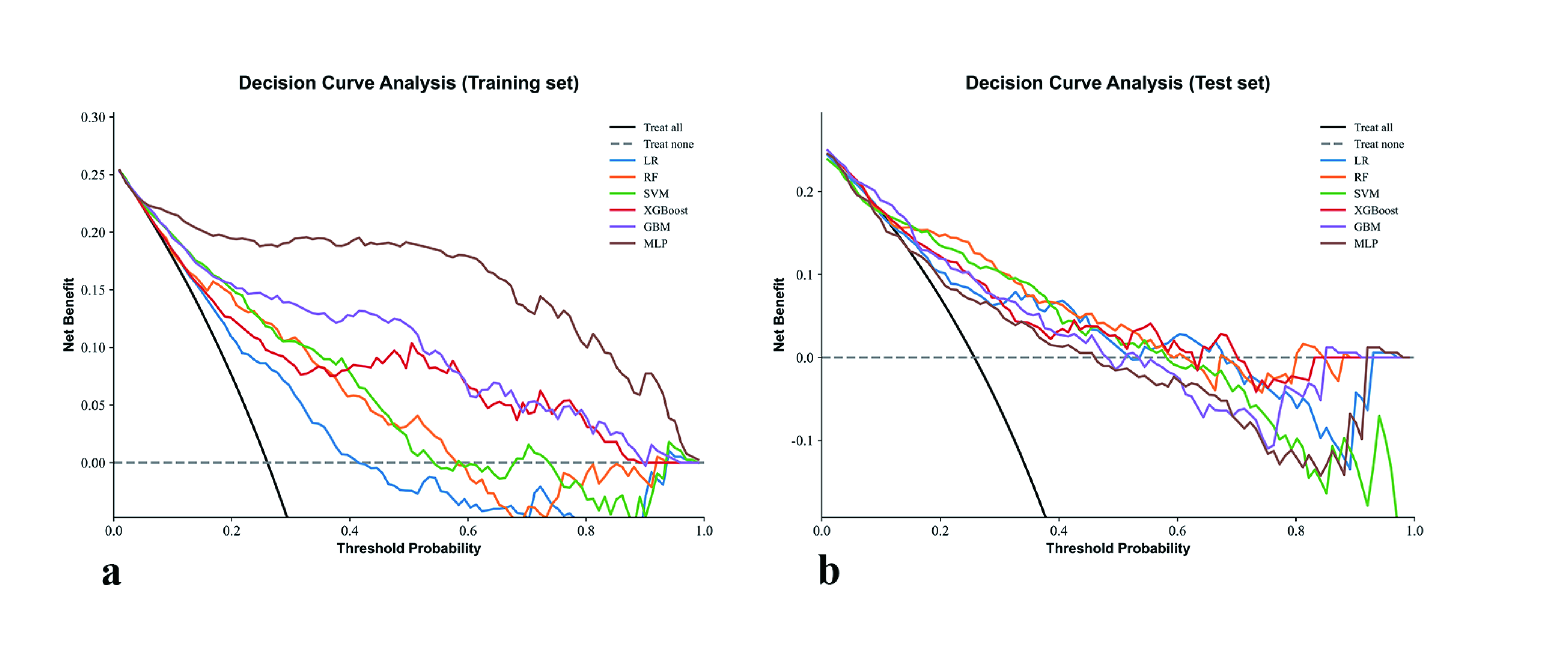

Supplement: Supplementary file 3 [file Image3.tif]

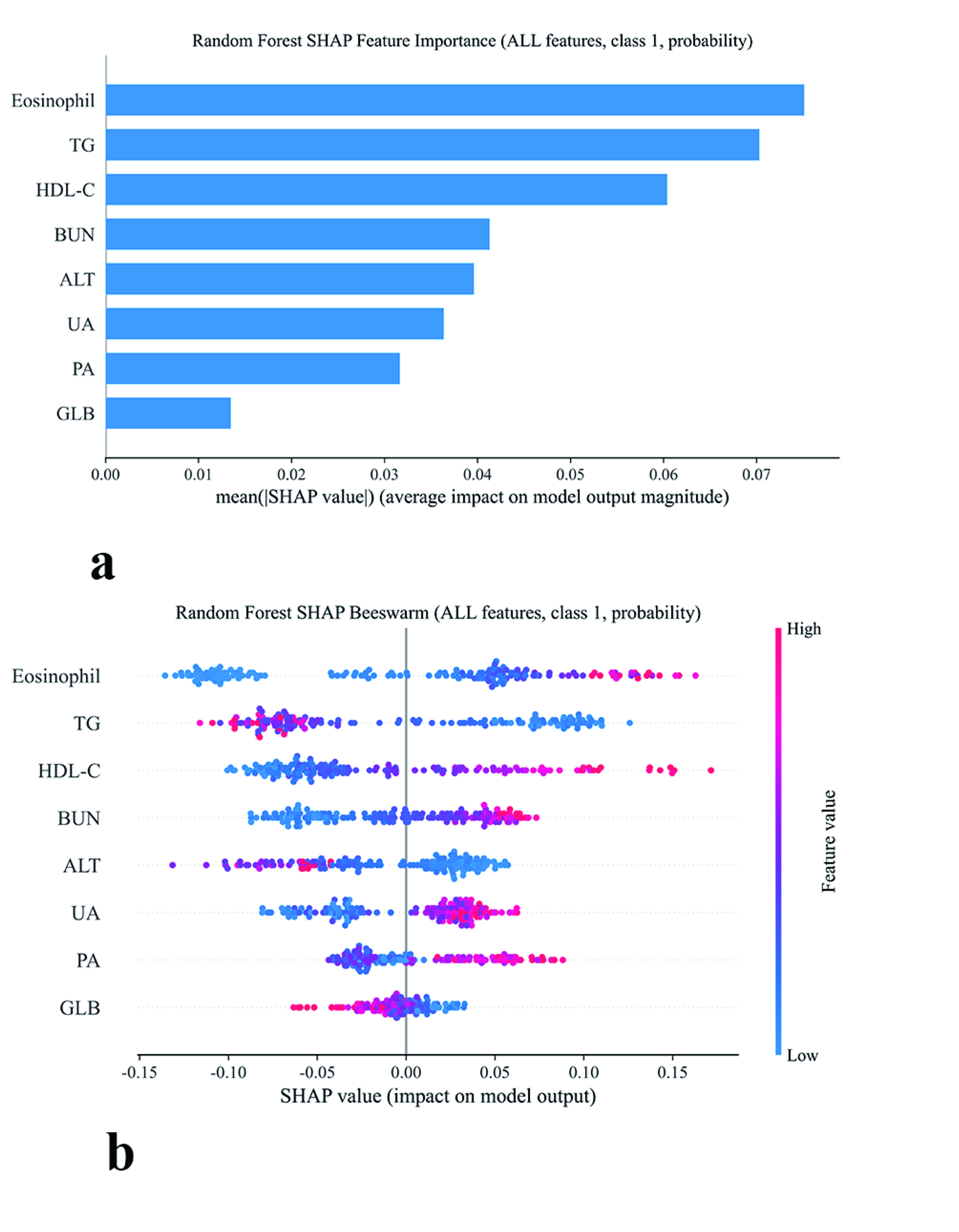

Supplement: Supplementary file 4 [file Image4.tif]
